# Transactions of the Medical Association of Southern Central New York

**Published:** 1854-08

**Authors:** 


*Transactions of the Medical Association of Southern Central New York*, at the Eighth Annual Meeting, held at Homer, June 4, 1851. From the Society.

THE Association of Southern-Central New York, embraces many members with whom we are personally acquainted: and we are glad to see by their annual volume of Transactions that they are not only prospering in, but also zealously laboring to improve their profession. The number of Transactions before us contains 120 pages, filled with the following articles, viz., The "President's Address," "Opium and Sulphate of Quinine as Remedial Agents," "An Essay on Medical Education," "Circulation of the Vital Fluids in Animals." "Sub acute Pleurisy." "An Essay on the Depraved General Health sometimes occurring before Parturition, and its consequences," "Hypochondriasis terminating in death." "A case of Cyranche Trachealis, complicated with inflammation and ulceration of the Soft Palate." "Report of a case of Puerpural Convulsions." "Traumatic Phlebitis." "Sloughing of the Caput Colli." "Hypertrophy of the Heart, with Dilitation." "Cases of Typhoid Fever." "Report of Committee on Surgery of Chemung County." "Surgical Cases, by Dr. P. B Brooks, of Binghamton," "Report of the Committee on Endemics and Epidemics of Tompkins County," and the Proceedings of the Society.

The first of these papers is a well written address by Dr. F. Hyde, of Cortlandville, on the importance of a General Study of Anatomy and Physiology. The second paper is a short essay on the remedial uses of Opium and Quinine. The author presents no new views or important facts. The paper is chiefly characterised by an almost indiscriminate recommendation of Opium and Quinine in the treatment of inflammatory as well as febrile affections.

It is written by Dr. Nivison of Hector, N Y The third paper, on Medical Education, is from the pen of Dr. Daniel Holmes of Smithfield, Pa It is a well written Essay, and for the most part inculcates sound doctrines For improving the education of the profession, Dr. Holmes urges the practical adoption of the following propositions, viz :

"First. That there should be a more thorough preliminary education. Second. That the term of private office instructions be more thorough and extended: and third. An extended term of medical lectures, and that the colleges be more rigid in receiving and graduating students"

These are good propositions—the same indeed that have been urged by the American Medical Association from the day of its organization to the present time. But how shall they be realized in practice? It is easy to meet in Conventions and Associations and resolve, but who carries out the resolve in every day practical life? Does Dr. Holmes himself always do it? We fear not, for in this same address he speaks of a young man who applied to him, whose "*preliminary education was very limited,*" and yet he received him as a student and for four or five months continued to help him to gain admission into the profession. And yet he blames some school for receiving and graduating that same student.

We recollect the first young man that ever applied for admission into our office as a student On examination we found him destitute of a competent knowledge of the most common branches of education We refused to receive him He went directly to a neighboring practitioner of age and experience and gained an easy admission to his office as a pupil. Yet that practitioner was a man of high standing, and is now a good member of the same Association to whom Dr. Holmes directed his address.

The truth is there is a great deficiency in the feeling of *personal* responsibility on the part of each individual member of the profession; and until this deficiency is supplied—until the great mass of the profession are made to feel personally a keen sense of responsibility in reference to everything pertaining to the introduction of students into the profession, formal resolutions will remain as they have been, little better than idle forms.

We had intended to notice some of the other papers in this volume, especially that on "Sub-acute Pleurisy," by Dr. Henry S. West, of Binghamton ; but we have only room to commend it as a well written and valuable paper. D.

